# “Envy in the Feed”: Pattern-Specific Social-Media Predictors of Depression and Anxiety among Chinese College Students

**DOI:** 10.1192/j.eurpsy.2026.12063

**Published:** 2026-07-31

**Authors:** X. Fan, X. Jin, T. Lyu, X. Hou, W. Guo, S. Wang

**Affiliations:** 1 Department of Clinical Psychology; 2 Department of Geriatrics; 3 Teaching Management Department; 4 Research Management Department, The Sixth Affiliated Hospital of Kunming Medical University, Yuxi, China

## Abstract

**Introduction:**

Depression and anxiety are prevalent mental health concerns among Chinese college students. Algorithm-curated “highlight reels” on WeChat, Weibo and TikTok amplify social comparison and fear of missing out (FOMO), both associated with heightened risk of depression and anxiety. But previous work rarely tested whether specific use patterns predict mood symptoms while adjusting for education level or social support.

**Objectives:**

To examine the association between social media use and symptoms of depression and anxiety, and to derive parsimonious, pattern-specific algorithms that quantify the risk of depression and anxiety from social-media behaviors.

**Methods:**

Nationwide cross-sectional online survey (March 2024–May 2025) of 1,030 students (16–30 y) recruited through student-union mailing lists in China; 93.6 % response rate; 67.9 % women; mean age 20.8 ± 2.3 y. **Measures:** Depression & Anxiety: Zung Self-rating Depression Scale (SDS, Chinese cut-off: ≥53) and Self-rating Anxiety Scale (SAS, ≥50). Social-media inventory: Social Network Site Intensity Scale (SNSIS, 6–30), Active/Passive Use Sub-scales (5-15), Social Media Envy Scale (2–10), Bergen Social-Media Addiction Scale (6-30), and self-reported daily duration (h). Covariates: Age, gender (0=male, 1= female), educational level (1=college, 2=bachelor, 3=master, 4=doctoral), and social support rating scale (SSRS).

**Results:**

Overall, 51.4% of participants screened positive for depression (11.3% moderate, 5.6% severe) and 33.1% for anxiety (8.3% moderate, 7.7% severe). Median daily social-media exposure was 6 h (IQR 5–10). Backward-LR logistic models identified a 4-item composite (SNS intensity, passive use, education, gender) that predicted depression with AUC = 0.67, and a 3-item composite (envy tendency, addiction, social support) that predicted anxiety with AUC = 0.78; both AUCs were superior to any single social-media metric (DeLong’s test, all p < 0.05, **Figure 1-2**). Final prediction equations (Nagelkerke R²): Depression: logit(p) = −3.41 + 0.051 SNS-intensity + 0.131 passive-use + 0.71 education + 0.25 gender; R² = 0.121. Anxiety: logit(p) = −2.81 + 0.116 envy/FOMO + 0.164 addiction − 0.036 social-support; R² = 0.285. Structural equation modeling further showed that FOMO mediated 41 % of the effect of SNS intensity on depression and 52 % on anxiety (standardized paths: a = 0.50, b = 0.16 and 0.31, respectively), indicating that envy in the feed is a key explanatory pathway (** Figure 3**).

**Image 1:**
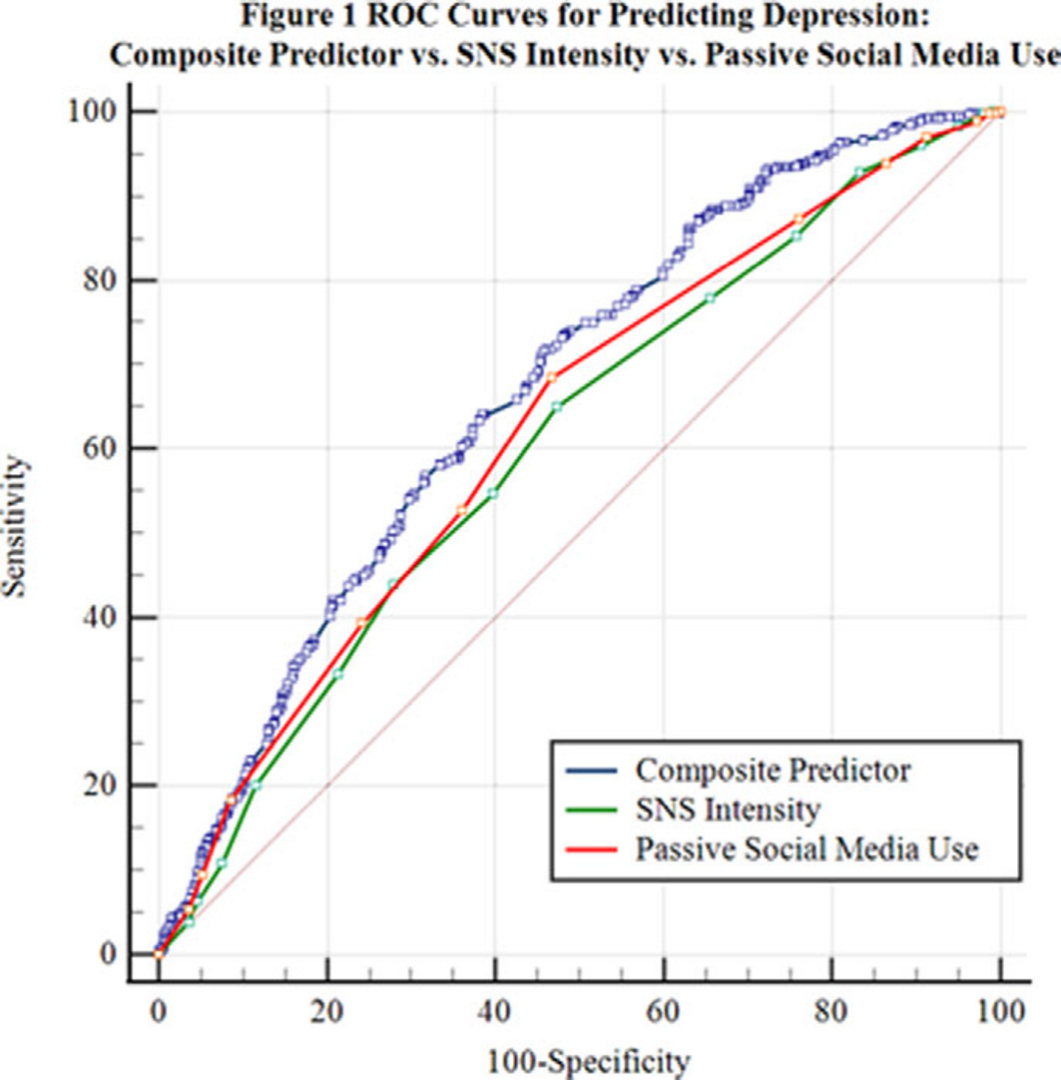
Image 1: Long description.

**Image 2:**
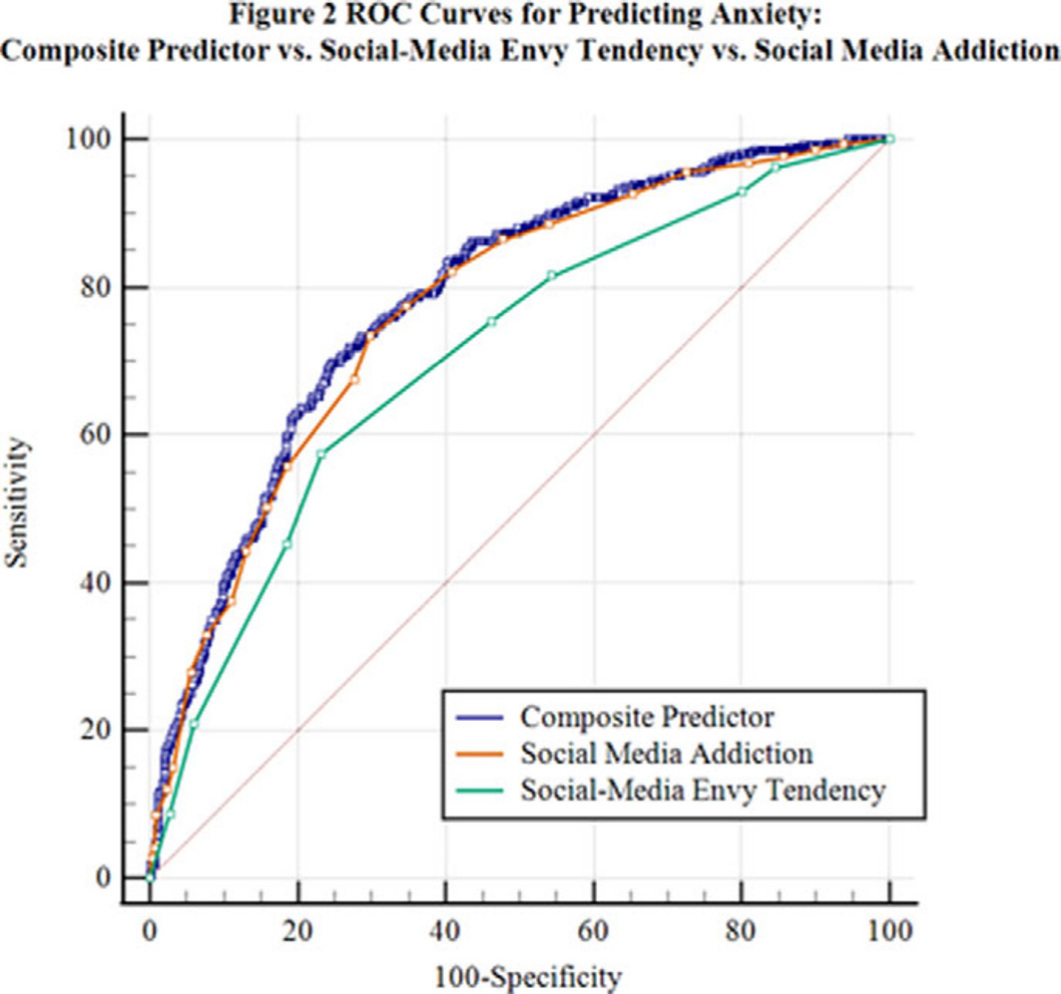
Image 2: Long description.

**Image 3:**
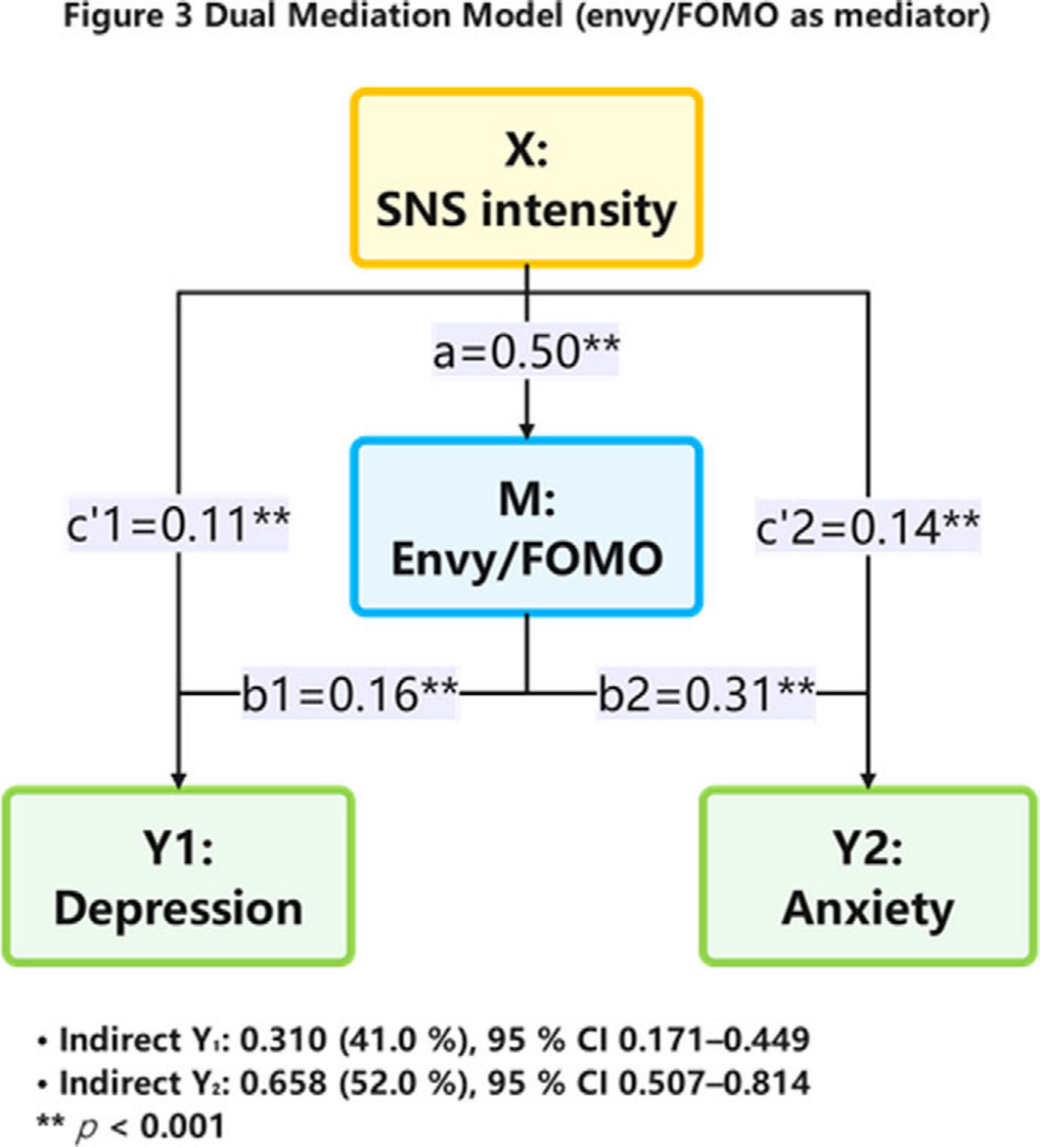
Image 3: Long description.

**Conclusions:**

In the large sample of Chinese college students, depression and anxiety symptoms are prevalent. A brief set of social-media metrics provides valid and parsimonious screening tools for depression and anxiety among Chinese college students. FOMO is a key modifiable pathway for digital interventions. **Founding:** The scientific research funds of Yunnan Provincial Department of Education (Grant No. 2023J0306).

**Disclosure of Interest:**

None Declared

## Long descriptions

### Image 1: Long description

A line graph showing Receiver Operating Characteristic curves. The Y-axis is labeled Sensitivity and the X-axis is labeled 100-Specificity, both ranging from 0 to 100. A thin diagonal pink line represents the chance level from 0,0 to 100,100.

Three data curves are plotted:

* Composite Predictor: A dark blue line with circular markers. It is the uppermost curve, showing the highest sensitivity across most of the specificity range. It follows a jagged upward path, reaching approximately 70 sensitivity at 50 on the X-axis.

* S N S Intensity: A green line with square markers. It sits below the blue line but above the red line for most of the graph. It shows a more linear increase compared to the blue curve.

* Passive Social Media Use: A red line with diamond markers. It is the lowest of the three predictive curves, tracking closely to the green line but generally staying slightly below it until they converge near the top-right.

A legend in the bottom-right quadrant identifies the three colored lines. All three curves perform better than the diagonal chance line.

Navigate back to Image 1:.

### Image 2: Long description

A Receiver Operating Characteristic R O C line graph. The Y-axis is labeled Sensitivity and ranges from 0 to 100. The X-axis is labeled 100-Specificity and ranges from 0 to 100. A diagonal red reference line runs from the origin 0,0 to the top-right corner 100,100.

Three data curves are plotted above the reference line:

* Composite Predictor: A dark blue line with square markers. It shows the highest performance, curving sharply toward the top-left corner, reaching approximately 60 sensitivity at 20 on the X-axis and 90 sensitivity at 50 on the X-axis.

* Social Media Addiction: An orange line with diamond markers. It follows a similar path to the Composite Predictor but remains slightly lower, particularly between 20 and 60 on the X-axis.

* Social-Media Envy Tendency: A green line with circular markers. This curve is the lowest of the three, showing a more gradual slope, reaching approximately 60 sensitivity at 30 on the X-axis and 80 sensitivity at 60 on the X-axis.

A legend in the bottom-right quadrant identifies the three lines by color and marker type.

Navigate back to Image 2:.

### Image 3: Long description

A flowchart illustrating a dual mediation model.

At the top is a yellow box labeled X: S N S intensity.

Three paths descend from X:

1. A central vertical arrow points to a blue box labeled M: Envy/FOMO. This path is labeled a = 0.50 with two asterisks.

2. A left vertical path labeled c'1 = 0.11 with two asterisks points to a green box labeled Y1: Depression.

3. A right vertical path labeled c'2 = 0.14 with two asterisks points to a green box labeled Y2: Anxiety.

From the mediator box M, two horizontal lines branch out to meet the vertical paths for Y1 and Y2:

- The path to Y1 is labeled b1 = 0.16 with two asterisks.

- The path to Y2 is labeled b2 = 0.31 with two asterisks.

Below the diagram, statistical data is listed:

- Indirect Y sub 1: 0.310 (41.0 percent), 95 percent C I 0.171–0.449.

- Indirect Y sub 2: 0.658 (52.0 percent), 95 percent C I 0.507–0.814.

- Two asterisks indicate p < 0.001.

Navigate back to Image 3:.

